# Understanding the Implications of Peer Support for Families of Children With Neurodevelopmental and Intellectual Disabilities: A Scoping Review

**DOI:** 10.3389/fpubh.2021.719640

**Published:** 2021-11-23

**Authors:** Michelle Chakraborti, Mojgan Gitimoghaddam, William H. McKellin, Anton Rodney Miller, Jean-Paul Collet

**Affiliations:** ^1^Department of Pediatrics, Faculty of Medicine, University of British Columbia, Vancouver, BC, Canada; ^2^Department of Anthropology, University of British Columbia, Vancouver, BC, Canada

**Keywords:** peer support network, caregiver, intellectual disabilities (ID), neurodevelopmental and intellectual disabilities, caregiver health, autism (ASD), families of children with neurodevelopmental and intellectual disabilities, neurodevelopmental disability

## Abstract

**Background:** Families are integrally involved in day-to-day caregiving of children with neurodevelopmental and intellectual disabilities (NDID). Given the widespread and increasing prevalence of children with NDID and the impact of family caregiving on psychological, social, and economic implications for both the child and family, understanding and supporting these families is an important public health concern.

**Objective:** We conducted a scoping review on peer support networks to understand their implications on families. Considering increasing prevalence of NDID's, understanding the implications of existing networks is critical to improve and nurture future support networks that can complement and reduce the burden on existing formal support systems.

**Design:** A comprehensive search of multiple databases was conducted. Articles were screened by two reviewers and any disagreements were resolved by a third reviewer. We explored existing research on parent-to-parent peer support networks, which included networks that developed informally as well as those that involved a formal facilitator for the group interpersonal processes. There were no limits on the study design, date and setting of the articles. We included all research studies in English that included an identifier for (i) “peer support networks,” (ii) “children with neurodevelopmental and intellectual disabilities” and (iii) “family caregiver outcomes.”

**Results:** We identified 36 articles. Majority of the studies were conducted in North America, and were face to face networks. They included families of children with a wide range of NDIDs. Relevant information extracted from different studies highlighted peer support network characteristics and development process, needs of family caregivers attending these networks, factors affecting caregiver participation and the impact of peer support networks on family caregivers. These networks represent a way to strengthen family caregivers, developing resilience and social interactions. Family caregivers sharing similar experiences support one another and provide critical information to each other. Although results are encouraging, future studies incorporating improved study designs are needed to better evaluate the effectiveness of peer support networks. Furthermore, studies where peer support networks develop organically while the child is supported are warranted.

**Conclusion:** Although results obtained are encouraging, our findings support the need for further research studies of peer support networks with better designs and more detailed description of the factors involved in the development.

## Introduction

The World Health Organization's International Classification of Functioning, Disability and Health–Child and Youth version (ICF-CY) highlights the “family” as an essential contextual factor for child-care and development (1). For children and youth with neurodevelopmental and intellectual disabilities (NDID), impairments in the child's cognitive, behavioral, motor, and/or language functioning develop at birth or during early childhood. Families are therefore, the primary caregivers for children with NDID, responsible for ensuring continuity of care across their lifespan (2, 3). Caregiving is a normal part of parenting and studies confirm that raising children with NDID also leads to parents' profound sense of meaning, purpose, and personal growth, including a renewed sense of patience, purpose in life, and gratuity (4). However, the intensive level of care required by children with NDID due to their often unpredictable behavior and demands, makes caregiving by families a public health concern with multifaceted psychological, social, and economic implications for both the child and family (5, 6). Evidence illustrates families of children with NDID typically experience higher levels of psychological distress and lower levels of well-being compared to parents of children with other disabilities, and typically developing children (7, 8). One key social factor contributing to these negative consequences is the feeling of social isolation due to their child's disruptive behavior and being treated badly by strangers due to lack of understanding about the situation (9); combined with the time-consuming care tasks undertaken by these parents, families often choose social isolation over the frustrations of taking their children out in public.

Social support is an effective buffer against stressors including social isolation (10–12). Past research has also identified social support as an important resource that families utilize to learn coping strategies, gain informational resources and emotional support (13). Specifically, social support from parents or peers, also referred as “peer support networks,” provides mutual support between parents who share similar experiences (14). The benefits of peer support networks for families have been previously identified in caregiving populations as enabling members to share their feelings, providing a sense of belonging to the community, and enlarging their social networks to include other families who can lend a hand during difficult times (15). Despite the acknowledged importance of the value of peer support networks for many complex conditions, we did not find studies that have reviewed in depth, the literature solely focused on peer support networks for families of children with NDID. Particularly, for families of children with NDID, compared to children with other complex conditions, research illustrates that raising a child with NDID such as autism is stressful for parents and families (7, 8). Children often exhibit behaviors that are disruptive and hard to manage, and can create instability in the family's functioning and leave parents feeling socially isolated, as they may fear taking the child out in public lest they create a scene. Consequently, parents of children with NDID experience a higher level of stress than parents whose children have other disabilities (16, 17). Mothers, specifically have been found to have poorer mental health, poorer physical health, and lower quality of life when they are parenting a child with NDID as compared with mothers raising typically developing children or children with other health or developmental impairments (13, 18). Thus, due to the nature of the child's severity in disorder, the severity of consequences on parents of children with NDID are often different than parents of children with other complex conditions. Therefore, understanding the implications of peer support in families of children with NDID is critical as it highlights whether these informal support systems are a possibility and also whether they benefit this specific population. And more importantly, with the increasing prevalence of NDID's, understanding existing peer support networks is critical to improve and nurture the infrastructure for future support networks that can complement and reduce the burden on existing formal support systems.

To address the knowledge gap, we conducted a scoping review on peer support networks to understand their implications on families. The literature was mapped into (1) the characteristics of peer groups and their development process; (2) the characteristics and needs of families attending peer support networks; (3) the impact of peer support networks on families of children with NDID, and (4) factors affecting the participation of families in peer support networks.

## Method

### Search Strategy

This scoping review was conducted using the scoping review methodological framework by Arksey and O'Malley (19). This scoping review maps out existing research on peer support networks for families of children with NDID. In order to identify relevant studies, a comprehensive search for articles was conducted using multiple databases: Academic Search Complete, CINAHL Complete; CINAHL with Full Text, Education Source, ERIC, MEDLINE with Full Text, PsycARTICLES, PsycEXTRA, PsycINFO, Social Work Abstracts, and SPORTDiscus. The publication year was not restricted. We searched peer-reviewed literature and also scanned the references of the selected papers.

All searches included a combination of the terms representing (i) “peer support networks,” (ii) “children with neurodevelopmental and intellectual disabilities” and (iii) “family caregiver outcomes.” We defined (i) Peer support networks as the presence of a community of similar interests where parents of children with NDID come together (in person or virtually by computer or telephone) to share their experiences, provide emotional, informational and instrumental support as well as find answers to their questions. This is consistent with definitions used in published Cochrane reviews, with the additional specification that the knowledge possessed by the peer support networks is concrete, practical, and obtained from personal experience rather than formal training (20, 21). We also defined (ii) children and youth with neurodevelopmental and intellectual disabilities, as children having impairments in the cognitive, behavioral, motor, and/or language functioning, resulting in a variety of challenges associated with ambulation, information processing, self-regulation (e.g., self-injury and unpredictable behavior) and communication (22, 23). We selected the highly prevalent NDID's, including Autism Spectrum Disorder (ASD), Down Syndrome, Cerebral Palsy (CP), Learning disability, intellectual disability, fetal alcohol syndrome (FASD) and attention deficit hyperactivity disorder (ADHD). Family caregiver outcomes (iii) were defined as the well-being of caregivers who were the immediate family members that included parents and/or mother and/or father of children with neurodevelopmental and intellectual disabilities. Studies that reported on outcomes related to caregiver well-being were included. The concept of caregiver well-being, the primary intervention outcome, is slightly nebulous in nature. Therefore, we anticipated that included studies will evaluate a broad range of measures related to one or more of the following: psychological health, family functioning, family resilience, family quality of life. The relevant keywords used in each individual search is provided in Table 1. A second search was conducted using a fourth term, i.e., “family caregiver term”-parents and/or mother and/or father. This search was conducted to ensure networks for specific family member caregivers, that is mother, father and parents were included. The search terms were truncated when appropriate to ensure relevant papers were captured by the search. The search was conducted until August 2020

**Table 1:** Key terms applied to conduct the search.

**Peer support network terms**	**Family care-giver outcome terms**	**Children with NDID terms**
**KEY TERM COMBINATION1**		
(“FAMILY SUPPORT GROUPS” OR “SOCIAL SUPPORT NETWORK” OR “SOCIAL NETWORK” OR “SUPPORT GROUPS” OR “GROUP PARTICIPATION” OR “PEER SUPPORT GROUP” OR “PEER SUPPORT” OR “PEER NETWORK”)	(“FAMILY OUTCOMES” OR “FAMILY FUNCTIONING” OR “QUALITY OF LIFE” OR “WELL BEING” OR “RESILIENCE”)	(“FAMILIES OF CHILDREN WITH AUTISM” OR “FAMILIES OF CHILDREN WITH DOWN SYNDROME” OR “FAMILIES OF CHILDREN WITH CEREBRAL PALSY” OR “FAMILIES OF CHILDREN WITH FETAL ALCOHOL SPECTRUM DISORDER” OR “FAMILIES OF CHILDREN WITH INTELLECTUAL DISABILITY” OR “FAMILIES OF CHILDREN WITH LEARNING DISABILITY” OR “FAMILIES OF CHILDREN WITH AUTISM SPECTRUM DISORDER” OR “FAMILIES OF CHILDREN WITH ASPERGERS” OR “FAMILIES OF CHILDREN WITH NEURODEVELOPMENTAL DISABILITY”)
**Peer support network terms**	**Family care-giver outcome terms**	**Children with NDID terms**	**Family care-giver terms**
**KEY TERM COMBINATION 2**			
(“FAMILY SUPPORT GROUPS” OR “SOCIAL SUPPORT NETWORK” OR “SOCIAL NETWORK” OR “SUPPORT GROUPS” OR “GROUP PARTICIPATION” OR “PEER SUPPORT GROUP”)	(“FAMILY OUTCOMES” OR “FAMILY FUNCTIONING” OR “QUALITY OF LIFE” OR “WELL BEING” OR “RESILIENCE”)	(“CHILDREN WITH AUTISM” OR “CHILDREN WITH CEREBRAL PALSY” OR “CHILDREN WITH DOWN SYNDROME” OR “CHILDREN WITH INTELLECTUAL DISABILITY” OR “CHILDREN WITH LEARNING DISABILITY” OR “CHILDREN WITH NEURODEVELOPMENTAL DISABILITY” OR “CHILDREN WITH AUTISM SPECTRUM DISORDER” OR “ASPERGERS”)	“PARENTS” OR “MOTHER” OR “FATHER”

### Selection Process

Inclusion Criteria: For study selection, a step-by-step selection procedure was implemented to obtain the relevant articles. Studies presenting original research published in peer-reviewed journals in English were included. The purpose of the scoping review was to explore and understand the existing literature on peer support networks for family caregivers (i.e., parents and/or mother and/or father) of children with NDID. Interventions which utilized a formal or professional facilitator were included, provided the facilitator's role was to facilitate discussion rather than providing didactic instruction, active counseling or psycho-education. We included studies that focused on families of children and youth as the support requirements for families of adults with NDID may be different considering the different health and support services and resources available compared to families of children and youth.

Exclusion Criteria: We excluded papers focused on: (a) informal support networks between friends (who do not have a child with a NDID) and/or family and/or colleagues; (b) measurement of perceived social support between friends (who do not have a child with NDID) or family or colleagues [e.g., (24)]; (c) facilitated groups that involved training by professional facilitators [e.g., (25)]; (d) studies pertaining to siblings and grandparents as we were interested in understanding the impact on the immediate parental caregiver, and (e) letters to the editor, commentaries, conference abstracts, plenary lectures, magazines and news articles.

**Figure 1:**
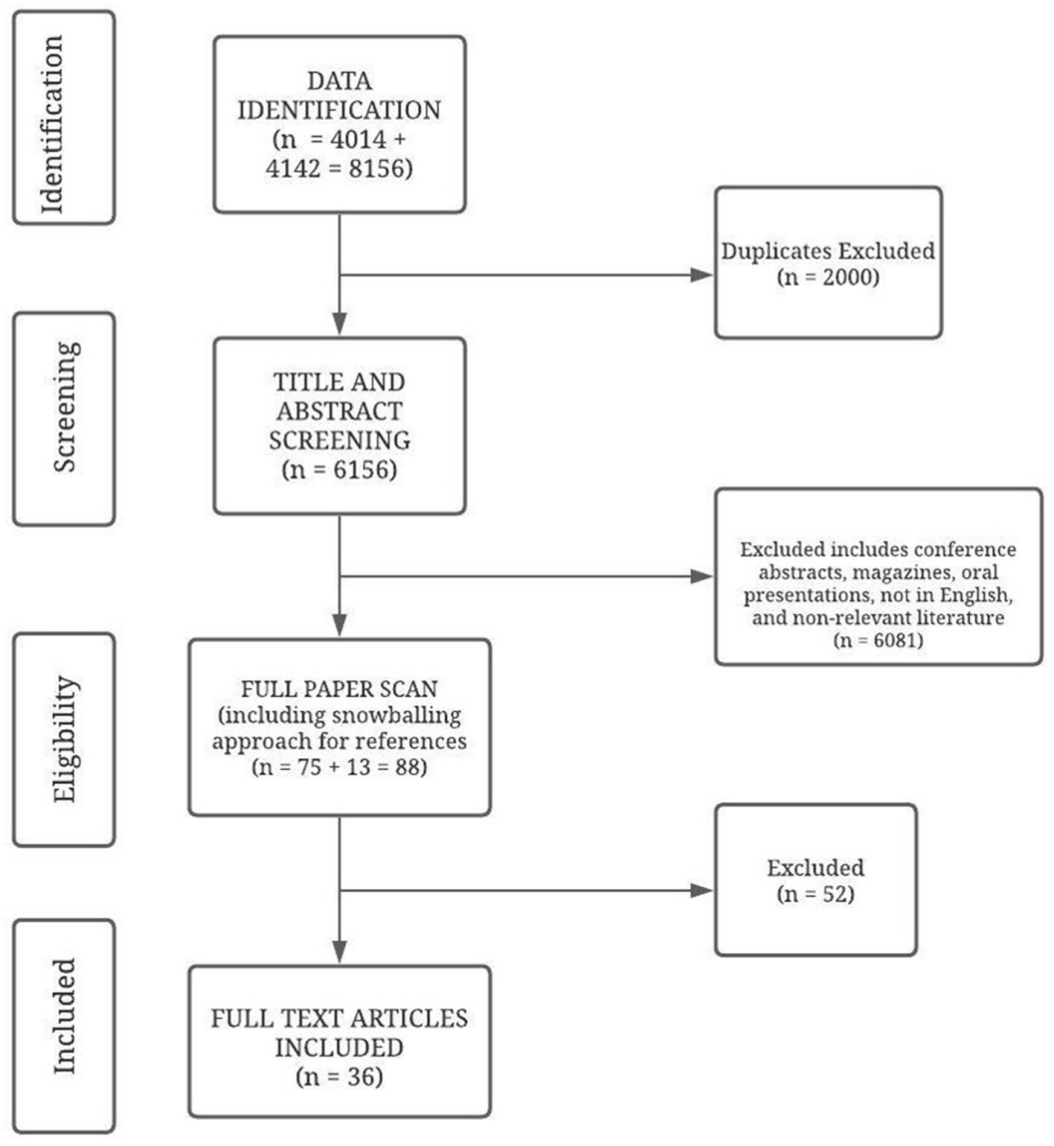
The Search Strategy.

Once all articles were retrieved, the duplicate articles were first removed. Then the remaining article titles and abstracts were screened and irrelevant articles removed. Titles and abstracts retrieved by the electronic searches were screened by two reviewers (MC and MG) using the inclusion/exclusion criteria. Disagreements were resolved by discussion with a third reviewer where necessary. The papers that did not meet the inclusion criteria were rejected. Next, full text copies of potentially relevant studies were obtained and assessed for inclusion using the criteria specified. Finally, the snowballing approach was implemented, in which the reference list of the selected articles were screened to ensure that all relevant studies were captured by the search. Any additional article found was further assessed for eligibility. The search details are provided in Figure 1.

### Quality Appraisal

Finally, as scoping studies provide a description of the literature, appraisal of the quality of evidence is generally not recommended in scoping reviews and therefore, was not formally conducted in this review (16).

## Results

### Search Results

Figure 1 outlines the procedure for article selection. The electronic search resulted in a total of 8,156 articles, with 4,014 articles from search 1 and 4,142 articles from search 2. Subsequently, duplicate articles were removed (*n* = 2,000) resulting in 6,156 articles. These articles were screened on the basis of title and abstracts. 6,081 papers were excluded. This included 5,552 articles that were not relevant to the focus, 123 articles not in English, 112 boos/book chapters, 92 magazine articles, 195 conference abstracts, 4 newsletters, 2 oral presentations and 1 letter to the editor. Following this exclusion, a total of 75 articles were obtained. These articles were read completely including references to ensure all relevant articles were included. Additionally, 13 articles were obtained while screening their references. Each of these 88 articles were read in detail by two coders (MC and MG) in order to verify relevance with regard to the objectives of the review. Articles that did not meet the inclusion criteria described in the selection process were rejected. 52 articles were excluded from the full paper scan due to two reasons, either the groups were referred as peer support groups but they provided didactic instruction or training such as counseling or psychoeducational groups; or the social networks/informal peer support groups involved friends or peers that did not have a child with NDID. This resulted in the final inclusion of 36 articles. All articles were accessed electronically.

Following the selection of studies, the next stage in the Arksey and O'Malley framework (19) involved charting the data. Using Arksey and O'Malley's charting criteria, we abstracted the data on the following criteria author(s), year of publication, study location, study objectives, study populations, intervention type, and comparator (if any), methodology and duration of the intervention, outcome measures and important results. In addition, the authors added another column “features of support groups” in order to provide information about the peer support group characteristics for each article in the table (where available). For details, please refer to Supplementary Table S1. Most of the peer support network research studies were conducted in North America, followed by Europe and Asia (Table 2); studies included perspectives of both parents' (*N* = 30), only mother (*N* = 4) or father (*N* = 2) (Table 3). Also, a majority of studies included parents of children with a wide range of NDID, rather than focusing on a single type of disability (Table 4), thus adopting a non-categorical approach (26).

**Table 2:** Division of papers based on country where research was conducted.

**Country**	**Number of studies**
Europe (Greece and Italy and UK)	8
Canada	8
USA	15
Australia	1
Asia (China and Taiwan)	3
Africa	1

**Table 3:** Family members participating in the study.

**Family member**	**Number of studies**
Parents (mother and fathers)	30
Only mothers	4
Only fathers	2

**Table 4:** Child disabilities represented in the studies.

**Neurodevelopmental and intellectual disabilities**	**Number of studies**
Mixed (e.g., ASD,ADHD, CP, severe learning difficulties, Spina bifida complex, additional needs with sensory issues, developmental delay, down syndrome, dyslexia, partial trisomy)	16
Autism spectrum disorder	14
Intellectual disability	3
Learning disability	1
ADHD	1
Down syndrome	1

### Literature Review Results

This is the final step of the Arksey and O'Malley framework where the information obtained is summarized and reported. The relevant information extracted from the different studies was mapped into the following categories: (1) characteristics of peer groups and their development process (2) characteristics and needs of families attending peer support networks; (3) impact of peer support networks on families of children with NDID, and (4) factors affecting the participation of families in peer support networks.

### Characteristics of Peer Support Groups and Their Development Process

Our study found that the peer support networks identified were either set-up by organizations (*N* = 22) or peer support networks designed by researchers (*N* = 14). No study described the spontaneous formation of networks formulated under the parents' leadership. The researcher-designed networks were implemented on the basis of community needs. For example, in the study by Kingsnorth et al., a parents' needs assessment was conducted, which guided the design for the peer support intervention for parents whose children had transitioned or were in the process of transitioning from youth to adults (34). The peer support networks under study either included parents only (*N* = 30) or networks that also provided child-care support while the parents attended the peer support network (*N* = 6). These peer networks involving child-care givers, e.g., (27) engaged the children in numerous activities such as arts and crafts. Their ability to engage children in different activities was the main focus of these networks as they allowed parents to focus on the group interactions.

The network size varied from a minimum of two individuals to a maximum of 150 individuals (28, 29). Interaction between parents at peer support networks involved either one on one interaction (*n* = 5), in which the trained parent volunteers, who were also parents of children with NDID, offered one-to-one support to their peers or support to groups of individuals (*n* = 31). These groups were led either by a parent peer leader or a professional facilitator whose role was limited to ensuring the efficient functioning of the group. Peer support networks were either conducted with face to face encounters (*N* = 31) or interacting virtually (*N* = 5) using email (*N* = 1) (30) or online encounters (*N* = 4) (31, 32). The duration and number of sessions parents met varied across studies. Some interventions only lasted 6-weeks with weekly sessions of 2-3 h each week (33);while others were conducted once a month with each session of 1-2 h per month (27, 34) or even three times a week during the school year from September to April (35).

The group's dynamics or the peer support development process were characterized by echoing, resonance, and mirroring patterns that not only favored the integration and cohesion of the group and promoted mutual identification but also de-individualized and normalized the experiences of the parents by invoking commonality between their situations to demonstrate that the parents had done nothing wrong (36).

### Characteristics and Needs of Parents Attending Peer Support Networks

The characteristics of parents attending peer support networks was the primary focus of two articles. On the basis of a survey administered to parents of children with ASD (*n* = 1,005), Mandell and Salzer (37) identified two-thirds of their sample to have participated in peer support networks in their lifetime. Parents involved in peer support networks had higher household incomes, educational attainments and were more likely to be in two-parent families.

Also, Mandell and Salzer (37) found participation was influenced by ethnicity, e.g., in studies in the US they are less likely to be African-American compared to Euro-American (*p* < 0.01). They also found that network participants were more likely to have older children and children with self-injurious behavior (*p* = 0.041)., sleep problems (*p* = 0.005)., and severe language deficits (*p* = 0.018) and were more likely to attend the peer support network if referred by a physician (*p* < 0.05). In another study on parent characteristics, Clifford and Minnes (38) discovered that parents of children with ASD (*n* = 149) attending a peer support network used more adaptive coping strategies such as seeking emotional and instrumental support than parents who never attended them (*p* < 0.001).

Furthermore, parents participating in peer support networks reported stronger belief in their benefits (*p* < 0.001), greater support from important others (*p* < 0.001) to participate in peer support networks, and fewer difficulties with participation (*p* = 0.01) than parents not presently participating. The authors identified two distinct groups of parents not presently participating in support groups: (a) parents who despite believing them to be beneficial, never tried them, because of difficulties associated with attendance such as the location, meeting time, and lack of child care, and (b) parents who attended them in the past, but found them not to be beneficial. Additionally, these latter participants reported less support from important others in choosing to participate.

We found only one paper that focused on the needs of parental participation in peer support networks. Papageorgiou and Kalyva (12) administered an open-ended questionnaire to families of children with ASD (*N* = 299); they identified four main reasons parents participated in support groups: (a) to be informed about the disorder (64.5%), (b) to receive practical help to develop their child's autonomy (19.5%), and (c) to meet and talk with other parents (8%), or (d) to benefit from counseling (3%). These needs reported by parents were influenced by their level of education. Parents with secondary school education wanted more practical support, as opposed to university educated parents who wanted more psychological support). Occupation was not correlated with the needs of the parents.

### The Impact of Support Groups on Families of Children With NDID

From the articles obtained in our search, we identified 25 articles with 4 quantitative studies, 17 qualitative studies and 4 mixed-method studies that measured and explored the impact of peer support networks. Quantitative studies reported various designs such as randomized controlled trials, quasi-experimental studies (pre- and post-test with follow-up), cross-sectional studies, and prospective cohort studies to understand the impact of peer support networks. The qualitative studies (including the qualitative part of the mixed-methods studies) used various techniques such as interviews, focus groups, and ethnographic observation. The interviews allowed the authors to capture the diverse experiences of individuals in these networks, while focus groups captured the dynamic interaction between group members and facilitators.

Numerous methodologies were adopted for the analysis of data; the constant comparative or grounded theory approach, ethnographic discursive approach and observation, interpretive descriptive thematic and content analysis, and case study and the framework method of analysis (For study details, please refer to Supplementary Table S1). The impact of peer support networks on the families of children with NDID, are categorized into seven themes. The process followed was similar to a thematic analysis. Two authors (MC and MG) read each article in detail and listed any attributes related to the impact of peer support networks. Then, each author compared the attributes they listed for each paper. Any disagreements were resolved by discussion with a third reviewer where necessary. Once the list of attributes were finalized, in subsequent meetings, through an iterative process, themes were proposed and discussed by the researchers until consensus regarding the theme structure was reached. The following themes were finalized and are discussed below: (a) creating a shared experience (b) promoting optimism and empowerment (c) learning (d) developing social opportunities (e) enhancing a sense of belonging (f) developing emotional and psychological well-being (g) developing advocacy skills and knowledge.

(a) Creating a shared experience: Creating a shared experience was the most common theme observed where both the individuals providing and receiving support understand each other as a result of having children with the same condition. This provided parents with the feeling of equality in their relationships with other parents (39) and positively reinforced them about their child (40), allowing them to speak honestly, to share and understand one another in a non-judgmental way (41). Parents found this as key to the successful functioning of a peer support network (28, 39). Moreover, sharing with others provided parents with hope (42), the ability to make meaning of their child's disability and validate their concerns (30, 34–36, 41).
  In addition, cultural similarity or belonging to the same ethnic groups, e.g., Chinese and Latina groups (27, 41) allowed parents to communicate comfortably in their own language, creating a comfortable bond among parents and making it easier for them to understand one another and overcome cultural problems such as stigmatization or shame in their own culture (43).
(b) Promoting optimism and empowerment: Peer support networks positively reinforced parents as demonstrated through participants' interviews (28, 39). Through networks, parents developed feelings of normalcy, received tips on how to manage day-to-day challenges, found security in having an available support, and benefited from helping others. Interviews with network participants also reported that participant parents felt empowered to handle problems (36, 39). Furthermore, parents reported that network participation allowed them to re-evaluate their parenting skills and their expectations of their children (44). The authors suggested that this could result from their increased sense of competence and self-efficacy after belonging to a peer support network (44). Furthermore, in a study by Kingsnorth et al., participation in a transition peer support network helped parents to envision a future for their child (30, 34). The repeated exposure to new ideas played a role in changing parents attitudes and generating hope for the future (44). However, in the multi-site mixed method RCT study by Singer et al. (45), the changes of scores on the empowerment scale were not statistically significant (*p* = 0.184), which is contrary to the qualitative data collected by the researchers. The authors indicated that the lack of robustness of the scale and therefore, the requirement of further psychometric evaluation of the scale.
(c) Learning: Discussions among parents within networks focused on knowledge about funding, caregiving, training opportunities and skills in navigating relevant social and healthcare systems (28, 34). Through interviews with parents of children with different NDID such as cerebral palsy and developmental delay, Ainbinder et al. reported that parental interactions initiated “new” learning among parents (39). Furthermore parents viewed this as an opportunity to learn from each other's mistakes resulting in the growth of parental adaptive coping skills (43). This experiential knowledge gained through interactions with other parents promoted awareness, hope, and an active plan for their child's future, especially during critical periods such as transitioning from adolescence to adulthood (27, 33, 34, 46).
(d) Developing social opportunities: The peer support networks allowed parents to interact and connect with one another (7, 33). These meaningful interactions were responsible for reducing social isolation, promoting social reinforcement and cognitive adaptation to emerging situations (37, 44, 47). Lo et al. in their study found that participants also reported that their gatherings not only strengthened their friendships with each other, but also provided their children with opportunities to develop friendships outside of school.
(e) Enhancing a sense of belonging: The relaxed non-judgmental interaction among parents was instrumental in creating a sense of belonging to their community (29). Consequently, parents cultivated friendly “family-like” connections with each other (27, 29, 43). Moreover parents in the studies described parent support meetings as an accepting and encouraging place where they felt welcome (41). For example, if a child had a behavior issue or meltdown at one of the Family Fun Day events, the parents felt that everyone understood because they had similar experiences with their own children (33).
(f) Developing emotional support and psychological well-being: The studies reported that seeking other parents of children with NDID for emotional support is often one of the main reasons parents participated in the networks (27, 30, 48, 49). These studies employed a number of different outcome measures. These included measures for psychological well-being and mental health [e.g., (47)]; quality of life [e.g., (40)]; depression and marital satisfaction [e.g., (47, 50)], coping [e.g., (51)], and stress, mood, anxiety, optimism [e.g., (31)]. Shapiro (51) in their study on mothers of children with NDID found that mothers who participated in a support group were less depressed than the mothers who did not (*p* < 0.01); perceived themselves as less burdened by their child than did non-participants (*p* = 0.05); and also tended to engage in more problem—solving coping strategies with their child than did non-participants (*p* = 0.04). However, there were no significant differences between participants and non-participants and other stress or coping scales.
  Similarly, the quantitative results obtained for other outcomes such as psychological well-being were not statistically significant in most of the cases. For example, the study by Shu and Lung (47) evaluating the effect of groups on psychological well-being (*n* = 27), found that despite the differences in the means of the psychological well-being scores between the intervention and control groups at the end of the program, the results were not statistically significant (*p* = 0.06).
  In the multi-site mixed method RCT study by Singer et al. (45), a statistical significant difference was observed between the intervention and control group on family strength and closeness (*p* = 0.003). Minimum coping scores suggested that parents attending the peer network supported their family strength and coping. In fact parents have also mentioned that groups became an important reference point in their lives by providing reassurance by merely knowing of the groups' existence (52).
(g) Developing advocacy skills: Researchers in several studies observed that parents became experts in providing solutions for their child with NDID, especially regarding community integration (27, 30, 46). However, parents also recognized the difficulty of influencing community changes without an organization behind them. Thus, being part of the group encouraged participants to advocate for the needs of their children and discouraged ineffective practices (41, 53, 54).

### Factors Affecting the Participation of Families in Peer Support Networks

Although seven papers discussed factors affecting participation in peer support networks, this was the primary research theme of only three articles (55). Participation of families in peer support networks was affected by the nature of the facilitators, parents and support organizations. A committed and motivated facilitator was critical for the optimal functioning of a group. In the study by Shilling et al. (55), interviews with parents participating in one peer support network identified that the facilitator's training (i.e., a peer parent) was essential to ensure they had updated knowledge in the area, a protocol for maintaining confidentiality and good listening skills.

Characteristics of the parents also influenced the group's functioning. As individuals differ in the amount and way they cope and share, the timing of support was an important factor. In their study, Shilling et al. (55) identified that peer support networks were beneficial for parents who were open and prepared to share with the other parent, while maintaining a degree of professionalism. Parental values or beliefs affected their suitability in the match of parents to groups [e.g., (30, 39, 44)]. For instance, there were divergent views on the matching of parents according to the cause of the child's disability, where some parents preferred to interact with families of children with the same disability [e.g., (39)], while in another study [e.g., (56)] the families felt the diversity allowed them to learn more and implement similarities. Hammarberg et al. (56) observed that the child's disability severity did not affect parents in the study, instead they appreciated the diversity and being understood by peers.

The organization of child care is also important to facilitate family networking, especially when caregiving for the child is offered during the networking time (27, 35, 52, 57). Hammarberg et al., (56) showed that the presence of playful helpers who provided childcare, while the parents attended the support groups, allowed parents to focus on their networking activities Barriers to network participation were also identified. These included the unavailability of child care, transport issues, various competing families responsibilities, employment commitments and fatigue that prevented parents from participating in every session (35). Some parents expressed additional challenges to attending peer support networks including the overload of information and the emotional nature of the discussions which were overwhelming at times (34). Online networks, although convenient and flexible in terms of execution and attendance, raised concerns among some parents about confidentiality and the exchange of unreliable information (7). Another major point of concern identified by parents participating in research-based group support studies was that the support did not expand beyond the end of study (27, 28, 34).

## Discussion

Research evidence suggests these families of children with NDID experience more negative psychological outcomes than families of children with other disabilities or those having a child with normal development (58). Furthermore, high levels of parenting stress are often associated with poor outcomes in behavioral interventions for their children (59). Thus, it has been well-recognized that supporting the family is essential not only for the caregiver, but for the child's well-being as well (60, 61).

Numerous interventions have been proposed to support families of children with NDID. Support strengthens parents, provides them with emotional and information support and helps families to socially integrate within the community, thereby reducing feelings of social isolation and providing a sense of belonging. In this review we focused on peer support networks formed by parents of children with NDID. We excluded professional led networks that provides didactic instruction or training such as psychoeducational or counseling or therapy groups, as the group's dynamic is different compared to peer support networks (62). To our knowledge, this is the first review of the literature that focuses specifically on parents of children with NDID. Our review led to the inclusion of 36 articles that represented research in 6 countries suggesting that peer support groups or networks are used worldwide by families of children with NDID.

Regarding characteristics of peer groups, Our results show that most network meetings were designed for face-to-face settings (31/36) compared to online (5/36). However, online group meetings have been studied more recently and are becoming more popular, especially with the growth of secure online platforms. These online networks have the ability to overcome some disadvantages of traditional face to face networks such as convenient times or distance, thereby allowing more people to participate from home. However, as this medium becomes popular due to its convenience, more studies are needed to understand the impact in further detail.

In addition to the medium of the networks, we also observed the duration of the network experience varied across studies, from once per week for 6 weeks to thrice per week for 9 months. It is interesting that, irrespective of duration, the families found the peer support networks helpful, but some parents worried about the support termination in context of a study. More studies are needed to understand what parents receive from peer support networks over different times and the parents' change in needs over time. This may help to develop networks that can support different parental needs over different time periods. Similarly, the density of exposure requires more in-depth study. If we assume that parents interact with their peers to the extent that they perceive it useful, it would be interesting to understand the specific needs that lead parents to meet three times a week vs. only once a month. Perceived need is certainly what leads most parents to participate in studies; for instance Ireys et al. (63) illustrated that compared to the mothers that were randomized, the mothers who self-selected themselves to not be randomized to access the group support, documented as having fewer psychological symptoms and a higher perception of their available social support.

Regarding peer support networks' organization, we found that those networks that offer child care were the most beneficial for the parents to overcome attendance barriers for parents. Although only six studies were designed in this context, (27, 33, 35, 41, 52, 56, 57) they all had a positive impact on the family outcomes. Taking care of the child may be a favorable way to facilitate peer support networks and may improve parental attendance at the activity sites. For example, in one case it was compulsory for the parents to bring their child to the summer camp for activities [e.g., (33)] or at the group meeting location [e.g., (56)]. Parents perceive child-care as a helpful idea that could benefit the child as well. Additionally, these groups foster the development of friendship among children within the playgroups. Research studies in this direction, where peer support networks develop organically when the child is supported for various activities are warranted.

Regarding parents' characteristics, we found that parents involved in peer support networks had higher household incomes and educational attainments, were more likely to be in two-parent families, and more likely to be Euro-American. In fact, the latter finding is consistent with the literature on support group use in other populations such as parents of children with type 1 diabetes [e.g., (64)] where the majority participants were Euro-American. This may be related to the cultural homogeneity that is required for good communication of personal worries (37). This is confirmed by studies on ethnic groups such as Chinese parent support groups that show parents' preference for “ethnic groups” for better communication. Mandell and Salzer (37) showed that participants in most studies were more likely to have elder children with higher severity such as self-injurious behavior, sleep problems, and severe language deficits; which may represent the special needs of parents with severe conditions or at critical time (puberty or transition to adulthood) (12). Although, the studies in our search included both parents of children and youth, further studies are needed to examine the benefits of peer support for families with children in different age groups and functional levels. This will help to understand the needs of parents having children in different age groups, which are critical periods of development, adaptation and adjustment for the child and the parents. Furthermore, comparative analyses between peer support networks and formal instruction support like psycho-education for different developmental and age levels, may help to understand which groups may benefit most from the different types of support.

Regarding the network impact, while analyzing the information, we found that shared experience among families was key to network formation, providing them with an avenue for learning, social integration, optimism and empowerment. The studies carried out were either quantitative, qualitative or mixed-methods in design. Interviews suggested that peer support networks were effective when participants perceived sameness among each other, fostering a shared identity. Consequently, this provided positive reinforcement in families such as reduced isolation, emotional and informational support, feeling of belonging to a new community, hence reduced stress and improved skills to better adapt to the situation groups [e.g., (28, 39, 41)]. These themes are consistent with the literature on self-help (65) and social comparison theory. Thoit proposed that self-help programs work via the perceived sameness of experience that members share. The social support offered by self-help groups serves as an extension of individual coping efforts. According to Thoit's theory, individuals' efforts to cope with challenging circumstances are enhanced and promoted through the modeling and practical advice offered by other group members (30, 45).

Although, encouraging, it is critical to keep in mind these study results report the parents' experience of those who attended the groups from the beginning to the end and therefore, were more likely satisfied than those who left earlier. This “prevalence bias” is a general concern of all these studies whereby those involved actively in a support service are likely to have an optimistic view for it; this places restrictions on the inferences that can be drawn from the review. Thus, in order to overcome this, future studies, should include a prospective follow up of each individual with careful recording of the reasons for leaving the peer support network.

Quantitative studies were mainly exploratory with small sample size. These studies showed similar benefits as qualitative studies regarding psychological well-being, coping and empowerment; however, because of small sample size, most differences were not statistically significant. Although encouraging, these results deserve cautious interpretation because of possible biases such as self-selection bias or the Hawthorne effect. Causal inference is another issue as association between variables, even if statistically significant, does not establish causation. For instance, people who have good psychological and mental health may be those that are attending the support group until the end. Therefore, in order to better understand the efficacy of the peer support networks, we need more robust study designs, such as randomized pragmatic trials with larger sample sizes and careful follow-up of each participant. The 4 mixed method studies provided a broader and deeper understanding of the outcomes and enhanced the results' validity [e.g., (40)]. Mixed-method designed studies are certainly worth considering in future studies; even the design of randomized trials will better evaluate the effectiveness of support groups for the families (66).

Another limitation of the published studies is the possible contamination by co-interventions, e.g., other family supports, organizational support obtained by parents on their own. The impact of these co-interventions is difficult to assess and represent a serious threat to the results' validity as we do not know precisely which factor leads parents to perceive a benefit. Thus, future studies need to better assess the concomitant interventions to better understand the specific benefits of the peer support network.

## Limitations

Although we conducted a comprehensive search using key databases and snowball searches, it is possible that some papers may not have been found. Furthermore, we did not include gray literature or non-peer reviewed literature in this scoping review because we could not assess their quality. Furthermore, a risk of publication bias is possible if some studies that did not find positive results have not been published. We believe such a risk to be low in context of assessing the role of social networks to support families with a child with NDID.

The inherent nature of a scoping review involves in understanding the nature and characteristics of peer support groups provided by a summary of the evidence from a limited range of publications. Therefore, in future, a comprehensive systematic review can be carried out that involves evaluation of the studies' validity and possible bias evaluation.

## Conclusion

In conclusion, providing optimal care for the child is a family affair and therefore, supporting the well-being of the family caregivers is essential as it directly impacts the well-being of the child. Peer support networks represent a way to strengthen families, building resilience and developing social interactions. Family members who share similar experience can support one another and provide critical information to each member. Future studies incorporating the recommendations mentioned above will help developing more adapted peer networks to support families in meeting their specific needs, and to better evaluate their effectiveness for the parents, the family and the child with NDID.

## Author Contributions

MC and J-PC contributed conception and design of the study. MC conducted the search, organized the database, and wrote the first draft of the manuscript. MC and MG (second reviewer) screened the articles obtained and any disagreement in the selection of articles was ruled out by a third reviewer, J-PC. J-PC, WM, and AM edited sections of the manuscript. All authors contributed to manuscript revision and read and approved the submitted version.

## Conflict of Interest

The authors declare that the research was conducted in the absence of any commercial or financial relationships that could be construed as a potential conflict of interest.

## Publisher's Note

All claims expressed in this article are solely those of the authors and do not necessarily represent those of their affiliated organizations, or those of the publisher, the editors and the reviewers. Any product that may be evaluated in this article, or claim that may be made by its manufacturer, is not guaranteed or endorsed by the publisher.
