# Supplementary material for: Understanding the Implications of Peer Support for Families of Children With Neurodevelopmental and Intellectual Disabilities: A Scoping Review
Source: Front Public Health. 2021 Nov 23;9:719640. doi: 10.3389/fpubh.2021.719640 (PMC8649771; doi:10.3389/fpubh.2021.719640)
Supplement: Supplementary file 1 [file Table_1.DOCX]

# **Supplementary Table S1: Summary of peer reviewed research articles on peer support networks for families of children with NDID**

| **First Author; Year; Country where study conducted** | **Study Objectives** | **Parent Population; Child's characteristics** | **N= (parent, control, intervention group)** | **Methodology; duration** | **Features of support groups** | **Measured Outcome** | **Important results** |
| --- | --- | --- | --- | --- | --- | --- | --- |
| Shu, B.  2005, Taiwan | To investigate the effect of peer support groups on the mental health and quality of life for mothers of children with autism | - mothers of children with autism (age between 30-51 years)  - children diagnosed with autism | 27, 19,8 | -quasi-experimental; pre and post test  -1 month follow up; 14 weeks | - face to face  -facilitated by psychiatric nurse who helped facilitate interaction among members | - Chinese health questionnaire (for psychological well-being)  - WHO quality of life questionnaire | - mental health did not significantly improve in the intervention compared to the control at the end of the first month and at follow up. However, employment status and subjective well-being affected mental health. Also, subjective well-being impacted quality of life.  - support group participation provided emotional support to the mothers. Moreover the presence of a support group served as a buffer to stress.  - the group leaders required continuous training in order to better support the caregivers |
| Santelli, B. 1995  USA | To explore the importance of the parent to parent support program as part of comprehensive family services | - parents participating in existing support groups;  - children with different disorders: developmental delay, down syndrome, mental retardation, autism, learning disability, visual impairment, CP , multiple disabilities, chronic illness, prematurity, chronic illness, prematurity, hearing impairment, and technology supported | 200 referred and 330 veteran parents | Two survey administered: one for referred parent and the other for serving parents | - face to face;  - pre-existing group 1:1 peer to peer  -referred parent matched with a veteran parent. |  | -the support groups provide referred parents with informational, emotional and other program supports (e.g. 24 h lines, family social events, group meetings for education etc.  -Similarly veteran parents were benefitted from the training they received |
| Elfert,M. 2015  Canada | To examine the impact of a support group for fathers of children with ASD | fathers of children with ASD (not be receiving psychotherapy or taking medication for mental health issues at the time of the study; Participants age range was 34–56 yrs  - children with autism (3-15 years) | 12  (Two groups of 6 fathers each) | -quasi-experimental; pre and post test,  - 8 weeks 2h sessions; with a 4-month follow-up  -Participants were assigned to one of the two groups based on their availability and geographic location (not random)  - During the eight weeks that Group 1 was active, Group 2 engaged in no formal activities related to the study. The day after Group 1 completed its final session, Group 2 commenced and ran for eight weeks. During these eight weeks that Group 2 was underway, Group 1 did not partake in any formal activities related to the study. Follow- up data was collected only from Group 1, four months after the conclusion of their group. | - face to face    -facilitated by the researcher but no didactic instruction provided | five standardized instruments:  - depression (Beck Depression Inventory-II)  - parenting stress (Parenting Stress Index – 4th Edition Short Form)  - marital satisfaction (Dyadic Adjustment Scale)  - optimism (Life Orientation Test – Revised)  - coping strategies (Ways of Coping Questionnaire ) | -Results indicated no significant changes ; however, participants strongly supported the importance of the support group experience |
| Clifford,T. 2013  Canada | To evaluate whether participation in an online parent support group affects parent reported perceived stress, symptoms of anxiety, depression, and positive perceptions of their child. | - Parents of children with ASD (33 to 53 years), control group (26 to 65 years )  - children diagnosed with autism (2 to 22 years); control group (3 to 17 years) | Finally (based on all test conducted, 25 parents (control) and 20 (intervention group) | -pre and post);  - 8-session support group, | - online parent support group designed by the researcher as a discussion group for parents  -facilitation from a counselling professional  - topics of discussion were based on parent suggestions | -parenting stress,  -positive perceptions,  -anxiety and depression symptoms  -participants completed a pre and post-group survey, | -No changes in parental well-being; however, the parents who participated in this online group reported being satisfied with the group and with the support received. |

| **First Author; Year; Country where study conducted** | **Study Objectives** | **Parent Population; Child's characteristics** | **n= PARENTS** | **Methodology; duration** | **Features of support groups** | **Important results** |
| --- | --- | --- | --- | --- | --- | --- |
| Kingsnorth, S.  2011  Canada | To explore the benefits, limitations and outcomes of a parent -led peer support group for families of children with special needs during the phase of service transition. | - parents of transition-age youth who are receiving augmentative communication support (7 mothers and 1 father)  - children with developmental and/or physical disabilities with a varied functional and communicative ability between 12-18 years | 8 | - Qualitative descriptive methods, focus groups were conducted in order to better understand the group dynamics; and observation  -11 Transition peer support group sessions over 1 year. Each session is 2 h | - face to face  - facilitated by experienced parent, experienced parent facilitated the interaction among families; 2-14 members in a group  - pilot program created for research purposes, | **-** emerging themes suggest the peer group experience benefited parents by providing them with awareness, active planning methods and knowledge based on parental experience about the transition process into adulthood |
| Law,M.  2002  Canada | To examine the effects of parent led support groups on parents of children with disabilities | -parents of children with disabilities (ages:20-49 years)  -children diagnosed with developmental problems: cerebral palsy, communication disorder syndrome, acquired brain injury Duchenne muscular dystrophy , global delay, autism, lupus, seizures, developmental problem behavior, developmental delay, seizures, hearing impairment, learning disorder, visual impairment ; ages: 0-17.9 years | 20 (from the nine parent led support groups were selected) | - open-ended interviews, observation of group meetings, and a review of support group documents | - face to face  - facilitated by an experienced parent  -pre-existing support program | -The interviews apprise the importance of parent led support groups: increasing skills, provide a sense of power and belonging. Moreover, participants interacted with each other and provided support and skills to deal with daily issues related to their child. |
| Shilling,V.  2015  UK | To explore the perceived outcomes for a one to one peer group for families of children with disabilities and the service providers | - parents and trained parent volunteers who were in contact with the Face2Face one-to-one befriending service during a 12-month period, and  -10 professionals in health, social care and education services who are responsible for providing referrals to families and are responsible for providing funding to organizations  -children of help seeking parents were diagnosed with: ASD, ADHD, CP, Auditory processing disorder,, Congenital heart disease, Currarino triad, Severe learning difficulties, Spina bifida (age range: 3.5–14) years WHILE expert parents had children diagnosed with CP, Complex additional needs with sensory issues, Developmental delay, Down syndrome, Dyslexia, Partial trisomy and Semantic pragmatic disorder (age range: 4–22) | 12 parents and 23 trained parent volunteers | -interviews and focus groups | - pre-existing program, parent is matched with a trained parent  - individualized support provided face to face  -facilitated by an experienced parent | -Shared experience was valued by all participant groups as a vital component of peer support.  - Key outcomes include reduced social isolation and emotional stability and personal growth.  -Likewise, by sharing information, the trained parents experienced positive outcomes by training, support being reciprocated and the feeling that they can help others.  -The negative outcomes included the emotional drain they felt by befriending the support receiving parents |
| Linder,R  1979  USA | To explore the benefits of a weekly support group | -parents of children with disabilities (ages:20-49 years)  -children with CP, muscle dystrophy or congenital cardiac anomaly | 5 mothers | -observation by researcher  -weekly meeting over 6 months | -face to face  -peer to peer support  groups initiated by researcher | -Mothers expressed the feeling of reassurance knowing that there was the group (a reference point) for them.  -The groups allow them to interact with each other. Also, the kids played with each other while the mothers participate in the group, and they look forward to the playdates |
| Mueller, T.G.  2009  USA | To explore the experiences of mothers in the support group for Latina mothers of children with disabilities | -Spanish speaking parents of children with disabilities CP, Down syndrome, Multiple disabilities(heart condition, cleft pallet, speech and language needs deaf, mobility issues, seizure disorder, Multiple physical and cognitive disabilities (visual impairment, cognitive delay, motor problems)  -The age of the children ranged from 4-16 years | 8 mothers | -semi-structured interviews were conducted  -monthly meeting throughout the year, with vacation during summer times | -face to face group  -expert led group (local community board member in order to facilitate the group peer to peer interaction  -informational seminars were also provided by experts;  -during the meeting time, child care for the children with disabilities and their nondisabled siblings were provided | -Emerging themes suggest the support group experience provided the Spanish speaking parents with a feeling of family among the members allowing them to discuss their concerns in their own language creating an environment of trust  -The group also contributed towards exchanging and receiving informational and emotional support  -The child-care provision was viewed as positive among the parents |
| Troester, J.D  2000  USA | To explore the experience of parents in a support group facilitated by researchers from a special education class | -parents of children with disabilities such as autism, learning disability, multiple and severe disabilities, hearing and emotional disabilities (children were between 6-24 years) | 17 parents in group; 8 parents participated in the evaluation | Qualitative analysis included observation, content analysis and evaluation by a form  -30 sessions in total; session offerings of one and a half hours, arranged on a biweekly basis and adjusted to the school year calenda | - face to face  - peer to peer support provided  - researcher initiated group group facilitated by the school social worker  -babysitters took care of children while the parents attended the group; | -Evaluation of the group based on a survey at the end of the group sessions suggest parents found the group beneficial as it helped them to become more vigilant and have their views corrected, informative, and they enjoyed the group experience.  -The content analysis illustrated that the main areas of discussion were about relationships, the emotional and physical needs of the children, community resources. -the parents felt they identified with each other. |
| Stallard,J  1993  UK | To explore the development and effects of a support group for parents of children with special needs | -mothers referred by professionals, these mothers have been experiencing difficulty in adapting to their child with special needs  -children were diagnosed with, learning disorder, SLD and mobility problems (15-30 months) | 22 parents; 5-8 in each group with 4 groups in total | - Qualitative analysis included observation, and evaluation by a form  -one session per week; 10-15 sessions in total | -face to face facilitated peer support group    - researcher designed group; facilitated by an expert psychologist and occupational therapist  -nurseries with trained nurses to babysit the children whose parents participate in the support group | -Group evaluation suggested the mothers found the group useful, they could discuss feelings, education and other benefits.  -The mothers group led to the formation of a fathers group. The fathers suggested it provided them with an opportunity such as to share their feelings, share problems with individuals in a similar position  - at the end of the groups, even though the parents suggested they would like more sessions in future, there were none after the group ended |
| McCabe, H  2013  China | To examine the benefits of two support groups: a) group for parents of young children verses b) parent with elder children with special needs | -group A: parents of children with autism and group B: parents with children with intellectual disability or mental health disabilities  -group A children were mostly 10 and under, except 5 families being 11 and above;  group B children were between the age of 20-33 | group A+B initial attendance was 42; number of total interviews from both groups=10 interviews | -semi-structured interviews were conducted one year after the support group was initiated,  -also, open ended questionnaires were administered during month 5 and 11 monthly sessions over the year qualitative | - face to face  - expert parent/researcher led, facilitated peer support group  -group organised by two chinese parents and researchers;  -for group A, parents left kids at home while for group B, parents brought children along | -Results from both groups suggest that the parents benefitted from the groups in terms of sharing of information, learning from others experience. -Interestingly, Group A parents requested more information to handle their children while  -group B parents were interested to know about more opportunities available for their children. |
| McCabe, H.  2008  China | To explore the experiences of participants before, during and after a short term support group program | -group A: parents of children with autism, attending the Autism Institute support group and group B: parents with children with autism attending the government run mental health centre  -children with autism (group A: 3-14,B: 4-7 years) | 43 parents in total from both groups | -semi-structured interviews  -group A: four, 11 week sessions per year (families are served in four small groups) while group B are individualized sessions | -group A is a facilitated expert led group while groupB is a one to one individualized peer support group  -face to face  -pre-existing groups; | -Emerging themes focus on the importance of these groups for parents (in two ways): as a means for sharing and learning from each other and, supporting and accepting each other |
| Lock,R.H.  2010  USA | To examine the family fun days experience as a venue for providing opportunities for parent’s support in addition to the children leisure activities | -parents of children with autism attending the family fun days (age range: 20-59 years)  -children with autism (age range:5 to 18 ears) | 25 families (23 mothers, 1 father and 1 grandmother) for the open end surveys; 11 participated in the interviews | -semistructured interviews, evaluative surveys and observation  -Family days expanded over 6 weeks in summer | -face to face  -peer to peer support provided; -initiated by the organization, based on parental requests; family days was initiated to bring the parents together | -Family fun days provided parents with the opportunity for interacting with each other and connect with on issues relating to their child.  - regarding parents time, parents felt more in control in the amount of time they spent in interacting and participating in fun activities with their children. Moreover, they did not feel pressured and looked forward to interacting with other parents.  -With an increase in the number of participants over the conventional support groups, these activities have the potential to attract families that avoid formal support groups to participate and interact with parents |
| Lo,L.  2010  USA | To explore the purpose and perceived benefits of Chinese families attending support groups | -Chinese parents with a child with disability who has participated in a support group  -children with autism, learning disability, cerebral palsy, hunter's syndrome and hard of hearing | 15 families | Semi-structured interviews | -families were recruited from two support groups hosted by a community organisation and a parent.  -The groups were parent led by a parent having a child with a learning disability -face to face interaction  -families were recruited from the 'same culture' support group | - the Chinese families participated in the Chinese support groups as they wanted to meet families who had the same cultural background and were experiencing the same 'cultural' pressures.  - participation in the group made them feel supported, provided the families with a sense of belonging, empowerment and informational support. Also, it helped to generate hope for the future |
| Huws,J.C.  2001  UK | To investigate the experiences of parents in an email discussion list | -parents in an email group concerning children with autism | 374 email addresses of parents were examined | symbolic interactionism methodology is used as as it recognizes the impact of interpretive processes such as the email discussions and social contexts such as the parents’daily life functioning in which meanings and actions are negotiated | -face to face  -online email group where pre-existing email group messages were analyzed | -the group provided an active social network for the parents.  - the email list allowed parents to reappraise their situation, make meaning and understand autism which supported them to adjust to changes, share experiences and understand and provide support |
| Frigerio,A  2016  Italy | To examine the dynamics of the interactions that occur within a self-help group for parents of children with ADHD | parents of children with ADHD attending a ADHD mutual support group | Over a 6-month period, the authors observed the meetings of a self-help group of parents of children diagnosed with ADHD. | - an ethnographic-discursive approach to observe the forms of interaction and collaboration exhibited among parents over 6 months  - the meetings were monthly, and each meeting lasted approximately 3 hours | -peer to peer support group  -face to face  -pre-existing groups;  -facilitator moderates the group | - Observation of the interactions among group members suggest that the production of a “homogeneous space” and a shared experience within the group allows parents to cope with issues and appraise themselves as good parents |
| Fine,M.  1983  Canada | To explore support groups for parents of children with downs syndrome and other disabilities | -from parents attending the Peel Infant Stimulation program, the parents were chosen by the program coordinators assessment, based on family functioning  children 2 years and under with down syndrome and multiple handicaps | 6 families per group (group size kept small to ensure all members get a chance for engaging with the group) | -observation over 6 weeks of a group created for research purposes | -expert facilitated (social worker and psychologist) peer support group  -face to face  -not a pre-existing group; the group created as a part of an infant program. It provides problem solving strategies for the parents to deal with their child | - feedback from the co-ordinators and the questionnaires filled by parents at the end of the program suggested the parent support program was beneficial for the parents as it allowed the parents to share information and emotions with each other |
| Carter,I  2009  Canada | To explore the experiences of parents of children with autism in an online self-help group | -parents with children with autism living in the Greater Toronto Areachildren with autism | 22 parents (17 mothers, 3 mother and father dyads, 1 mother and grandmother dyad, and 1 divorced father) | semi-structured interviews; thematic analysis | -Online pre-existing online peer support group | The emerging themes suggest, the online groups support families by providing them with information, opportunities to interact with each other and a tool for promoting advocacy among the members.  -The list of challenges included accessibility issues, lists of unknown and costly treatments, confidentiality issues. |
| Ainbinder, J. G.  1998  USA | To explore qualitatively the experiences of parents in the parent to parent support programs | -parents of children with special needs  -children with special needs  (age=1-16 years) special needs (non-categorical approach)= children with any developmental disabilities, learning disabilities, emotional needs or special health care needs- mild to severe diagnosis of CP, epilepsy, developmental delay, mental retardation. learning, hearing/vision deficits | 24 parents recruited were biological parents (23 mothers, 1 father) | -semi-structured telephone interview  -one on one, matched with trained parent;  parents were randomly assigned into two groups: either they were directly matched or waited for 8 weeks before matched | - peer to peer (one is to one match)  - pre-existing support program evaluated  -face to face -support group matched | -Parental attendance in the peer support networks resulted in favorable interaction and learning among parents  -successful match depends on a reliable supportive parent consisting: 1) similarity in condition 2) availability of individual 3) comparable conditions, allowing learning and growth 4) mutuality of support  -barriers to group formation : Parents busy schedule long distance between the parents involved negligent follow up by other parents |
| Aldersey,H.M.  2016  Congo,Africa | To explore the experience of parents in a self-help group | -parents of children with intellectual disability | 14 members (mothers, fathers, sister, grandmother) | -observation and semi-structured interviews peer support for eight weeks | -face to face support  - pre-existing support program evaluated  peer to peer support  -group leader is a experienced parent | -Through observation by the group members parents illuminated upon the importance of support provided by peers in the group- emotional, informational, material, instrumental and physical support |
| West,A  1998  UK | To pilot and explore a group for fathers of children with down syndrome | -parents of children with down syndrome  -children were aged from 14 weeks to 4.5 years | 4 fathers | -observation  (4 sessions, with each session for 1h) | -face to face support  - program developed by researcher to evaluate the importance of peer networks | -The researcher observed that the fathers gave and received emotional and informational support. Anger and anxiety were the predominant emotions expressed  -the researcher noticed the powerful group dynamics and the mentorship provided by the ‘senior’ fathers to the ‘junior’ fathers  -the fathers reported a positive experience for attending the group and would like to attend in future |
| Swanke,J  2013  USA | To explore the interest of mothers to participate in social networks through blogging | -parents of children with ASD | 25 mother bloggers | -observation by researcher (reading the blogs) | -online network | -the blogs demonstrated the emotional support blogger mothers provide to each other in their online world  -it provided them the ability to express themselves freely and separate from the physical world  -through blogs, the mothers provided each other with instrumental support as well such as feedback on parenting experiences, advocacy  -they referred to blogging as a therapeutic experience as it allowed mothers to communicate freely |

| **First Author; Year; Country where study conducted** | **Study Objectives** | **Parent Population; Child's characteristics** | **N (PARENTS)N (Control)N (INTERVENTION)** | **Methodology; duration** | **Features of support groups** | **Measured Outcomes** | **Important results** |
| --- | --- | --- | --- | --- | --- | --- | --- |
| Singer, George HS  1999  USA; conducted in five states: Kansas, New Hampshire, North Carolina, South Carolina, and Vermont | The study evaluated the effect of the multisite 'Parent to Parent' support programs for parents | -Parents, foster parents, or grand parents of children with a disability or chronic health conditions (examples include cerebral palsy, epilepsy, developmental delays, mental retardation, learning disabilities, hearing and/or vision deficits, and several chronic illnesses)*  Parents are not presently participating in the 'parent to parent' support programs, majority sample consisted of mothers and one father  -mean age of the children were 6.9 (intervention) and 7.7 (wait-list group) | 128 (Quantitative); 24 (Qualitative, randomly selected from the pool of participants categorized as those who found the group helpful verses those who did not in the quantitative part)72 ( waiting list control; these participants were provided with Parent to Parent support after the post-test) | -Mixed-methods: RCT, 2 months ; pre and post test comparison between intervention and control group;  -Qualitative: semi-structured interviews (emerging themes were identified using the constant-comparative method) | -peer to peer. Parents matched with a suitable trained mentor parentthe parent interacted with the mentor through 4 phone calls over 2 monthsPRE-EXISTING support program, 1:1 match with a mentor | -Cognitive adaptation (Kansas Inventory of Parental Perceptions scale);  -Empowerment (Family Empowerment Scale),  Coping (Parent Coping Efficacy Scale), progress made on a problem and helpfulness of the group | -parents using Parent to Parent for “non- urgent” help benefit from engaging with other parents by (a) coping better with their child and family’s situation, (b) view their condition with greater positivity and (c) able to progress on goals that are important and make a difference iin their lives  -Parents did not feel empowered by only a brief involvement with the program |
| Solomon, M.  2001  UK | The study examined the positive effect of support groups on parents of children with special needs | -six support groups were recruited for the study.  -Parents from these groups were recruited (the mean length of time for parents in these groups was 3.9 years). The sample constituted 52 mothers and 4 fathers  -mean age of the children was 9 years (range=1 to 26 years); the childrens condition included specific learning difficulty, dyspraxia, attention deficit disorder, or speech delay, a severe or profound mental or physical disability or both, autism spectrum disorder and moderate learning disabilities | -56 (Quantitative);  - 43 (Qualitative; | Mixed-methods: Quantitative: crosssectional study; Qualitative: focus group sessions (emerging themes were identified using the constant-comparative method) | -facilitated by a trained parent,  -the group interacted face to face  -meeting continued for 2 h  -pre-existing support program | -Overall helpfulness (session impacts scale), -satisfaction (client satisfaction questionnaire), -group social climate (group environment scale) | -From the quantitative data: parents significantly benefitted from their groups rating them high on helpfulness and satisfaction, cohesion and task orientation.  -From the qualitative data, parents' described an increased sense of control, sense of belonging to their community, and their experience of self change towards the situation |
| Bray,L.  2017  UK | To explore the impact of peer to peer support groups on the well -being of parents of children with special needs. | -parents of children attending the face to face support group  -children were diagnosed with ASD, Down’s syndrome,  Cerebral palsy, Attention Deficit Hyperactivity Disorder, Microcephaly, Fragile X , Foetal alcohol syndrome, Genetic disorders | -70 interviews (qualitative) were conducted and  -68 sets of questionnaires (quantitative) | -Mixed-methods  -prospective concurrent mixed method study design with baseline and follow-up data to map out any changes.  -Qualitative interview data and quantitative questionnaire data provided different but complementary sources of evidence and were afforded equal priority in the collection and analysis process | -peer to peer; one is to one match of befriender with befriendee  -face to face  -pre-existing support program | -General Health Questionnaire-12 (GHQ-12)(examines psychological distress and parental mental wellbeing), -Pediatric Inventory for Parents (PIP) (Assesses parental stress related), -Peds QLTM  -Family Impact Module ( Health Related Quality of Life and Family Functioning) | -The peer-to-peer parenting support created meaningful relationships for ‘befriendees’ for opportunities to thrive  - the Befrienders also thrived as a result of their interaction; through training, engaging with others and seeing possibilities for the near future  -The quantitative evidence is supported by the qualitative evidence; demonstrating improvements in emotional and psychosocial well-being.  -The befriendees transversed from a position of" ‘feeling lost’ and ‘struggling day by day’ towards a ‘better place.’" |
| Shapiro, J  1989  USA | To explore the relation between support group participation and meaning attribution to stress and depression levels in mothers | -mothers of children suffering from developmental delays, children with down syndrome, cerebral palsy, William syndrome, spina bifida, and developmental delay | -56 (34 already participated in a support group, 22 did not before) | -Mixed methods: Quantitative: questionnaires and semi-structured interviews  -All groups met approximately once a week for 1-1.5 hours | -face to face  -facilitated by a licensed psychologist  -pre-existing groups, | -The Center for Epidemiological Studies Depression Scale,  -The Coping with Stress Inventory,  -The Questionnaire on Resources and Stress | -mothers participating in support groups were less depressed than those who didn't; perceived as less burdened due to their child than those who did not take part and engaged in greater problem solving coping strategies with their child.  -no significant relation between participation and other stress or coping scales.  -Mothers rated high on the meaning scale, were positively correlated with decreased depression and stress (in terms of daily aspects of care). meaning was positively correlated with emotional coping and problem solving coping. However it is unclear whether support groups resulted in greater meaning or vice versa, higher meaning mothers received higher benefits in support groups |

| **First Author;**  **Year;**  **Country where study conducted** | **Study Objectives** | **Parent Population; Child's characteristics** | **N= (parent, control, intervention group)** | **Methodology; duration** | **Features of support groups** | **Important results** |
| --- | --- | --- | --- | --- | --- | --- |
| Shilling,V  2014  UK | To explore the factors (both facilitators and barriers) that contribute towards the development of a participants shared experience in a support group | -parents and trained parent volunteers who were in contact with the Face2Face one-to-one befriending service during a 12-month period, and 10 professionals in health, social care and education services who provide referrals to families and funding to organizations  -children of these parents were diagnosed with: ASD, ADHD, CP, Auditory processing disorder,, Congenital heart disease, Currarino triad,Severe learning difficulties, Spina bifida (age range: 3.5–14) years WHILE expert parents had children diagnosed with CP, Complex additional needs with sensory issues, Developmental delay, Down syndrome, Dyslexia, Partial trisomy and Semantic pragmatic disorder (age range: 4–22) | 12 parents and 23 trained parent volunteers | interviews | -face to face  -peer to peer match  -pre-existing support group | -Results suggest formal structures such as proper training of the matched parents, ongoing supervison and the rightful matching of the support receiving parent and befriender are important for the successful operation of a mutual support group |
| King,G.  2000  Canada | To explore the organizational characteristics and factors affecting the longevity of the self-help support groups | -parents of children with special needs were recruited from these nine groups (20 to 49 years)  -children CP, communication problems, various syndromes, and acquired brain injury (5%). Secondary development problems included developmental delay (70%), learning disorder, seizures, behavior problems, hearing impairments, and visual impairments. | 20 (10 mothers and 10 fathers) | -Qualitative (semi-structured interviews and observation of group meetings) | -face to face  -most groups held meetings once a month or less | -Emerging themes about important organizational characteristics determining the group longevity are effective leadership strategies, commmunity connections providing funds and the inclination of group members to change the activities to meet the shifting group needs. This allows the effective functioning of the groups over time. |
| Hammarberg,K  2014  Australia | To investigate the barriers and promoters for participation in peer support group programs | -parents/caregivers attending the group during the school year (25 to 45 years)  -children with special needs (autism, global developmental delay, down syndrome, cerebral palsy and respiratory and heart disease) | 20 parents | Qualitative (semi-structured interviews) | -Meetings of groups of 4–12 carers are facilitated by a trained group leader and assisted by a play helper.  -Group facilitators are workers qualified in disability, parenting or family support. Play helpers keep children, including under school-aged siblings, occupied with a range of activities while the parents engage in the group activities .  - In addition to reciprocal peer support, group members are offered information to help them navigate the disability funding and service systems and manage the daily functioning with a child with high and complex needs.  -Groups also have a modest budget to occasionally invite external speakers or people who offer services such as art therapy | -Good group facilitation, presence of play helpers, access to information and expertise, and the mutual support among members emerged as the most important promoters of group participation.  -Barriers included insufficient funding to run the program throughout the year, diversity in group members’ socio-economic position and severity of their childs disability. |

| **First Author; Year; Country where study conducted** | **Study Objectives** | **Parent Population; Child's characteristics** | **N (PARENTS)N (Control)N (INTERVENTION)** | **Methodology; duration** | **Features of support groups** | **Outcome measures** | **Important results** |
| --- | --- | --- | --- | --- | --- | --- | --- |
| Papageriou,V.  2010  Greece | To investigate the reason why parents attend support groups | parents of children with ASD attending support groups | 299 parents(72 fathers and 227 mothers; age ranging from 27 to 60 years) | Quantitative (open end questionnaire) | -face to face |  | -Most parents state that they participated in the groups as that they wanted to be informed about developments in the area of ASD, such as new therapies, wanted to receive support, engage with parents of children with similar disabilities, Also, the parents felt they needed pome informal psychological support that the group could provide  -They found that the types of needs reported by parents were influenced by their level of education, and gender and age of their children, but that there were no differences in the type of occupation of the parents. |
| Clifford,T.  2013  Canada | To examine the influence of beliefs about support groups, beliefs about significant others’ opinions about support groups, mood, coping style, and social support between individuals currently attending, previously attended and never attended support groups. | -parents of children with ASD (age range: 24 to 65 years) | 149 parents [based on their support group use: never used support groups (n = 36), past support group use (n = 37), and current support group use (n = 76)] | Quantitative (questionnaires) |  | -State Trait Anxiety Inventory,  -State-Trait Depression Scales,  -Kansas Inventory of Parental Perceptions,  -Types of Support Questionnaire, -Brief COPE,  -Views About Parent Support Groups Questionnaire | - the three groups differed significantly in their beliefs and attitudes about support groups and in their use of adaptive coping strategies; however, no significant differences in availability of peer support, anxiety or depression signs, or beliefs about control of ASD was observed.  - current support group users reported using more adaptive coping strategies than the other two groups. Past group users mentioned they did not find the groups as beneficial as current users, and those who never participated in peer groups reported difficulties with accessibility. |
| **e) Article on characteristics of families attending peer support networks** |  |  |  |  |  |  |  |
|  |  |  |  |  |  |  |  |
| Mandell,D.S  2007  USA | To examine the factors associated with support group participation | -family members (mothers, fathers and legal guardians) of children with autism (age range:23-70 years)  -children with autism (age range: 2-53 years) | 1005 participants | Quantitative (survey) |  |  | -Adjusted analyses suggest participation in a support group is associated with demographic characteristics such as child age, child sex, ethnicity, parent education and income.  -the surveys suggest parents of children who are self-injurious, have sleep and language problems are more likely to attend these groups, as well as parents who are referred by their consulting physician. |

* Please note: The paper by Singer et al. was obtained while scanning through the references of the paper J.Ainbinder (1998). The study by Ainbinder et al included a subset of the population from the study by Singer et al. Therefore, although the child’s diagnoses was not officially listed in the paper, I have listed the disabilities from the Ainbinder et al study under the Singer paper.
